# The Louisiana Community Oil Spill Survey (COSS) dataset

**DOI:** 10.1016/j.dib.2020.105390

**Published:** 2020-03-07

**Authors:** Michael R. Cope, Tim Slack, Troy C. Blanchard, Matthew R. Lee, Jorden E. Jackson

**Affiliations:** a Brigham Young University, United States; b Louisiana State University, United States

**Keywords:** Oil spill, Deepwater Horizon, BP, Disaster, Gulf of Mexico, Louisiana

## Abstract

This article presents an overview of the Louisiana Community Oil Spill Survey (COSS), the dataset used in “Community Sentiment following the Deepwater Horizon Oil Spill Disaster: A Test of Time, Systemic Community, and Corrosive Community Models” [1] as well as elsewhere [2–6]. The COSS, administered by the Louisiana State University's Public Policy Research Laboratory, consists of five waves of cross-sectional trend data attuned to the characteristics and effects of the 2010 BP Deepwater Horizon (BP-DH) oil spill on those coastal Louisiana residents most affected by the disaster. Respondents were randomly drawn from a list of nearly 6,000 households in the coastal Louisiana zip codes located in Lafourche Parish, Plaquemines Parish, Terrebonne Parish, and the community of Grand Isle. COSS data were initially collected in June 2010 when oil was still flowing from the wellhead, with additional data waves, collected in October 2010, April 2011, April 2012, and April 2013. The respective response rates were: June 2010, 20%; October 2010, 24%; April 2011, 25%; April 2012, 20%; and April 2013, 19%.

**Specifications table**

**Table:** 

**Subject**	Pollution
**Specific subject area**	The 2010 BP Deepwater Horizon oil spill, which occurred about 41 miles (66 km) off the coast of southeast Louisiana, USA.
**Type of data**	Tables
**How data were acquired**	RDD telephone survey
**Data format**	Raw
**Parameters for data collection**	Data were collected from a cross-section of Louisiana households in the coastal zipcodes of Plaquemines, Lafourche, and Terrebonne Parishes, and the community of Grand Isle. These areas were sampled due to their close proximity to the BP-DH spill.
**Description of data collection**	The COSS is a RDD telephone survey, sampling approximately 1000 of the 6000 Louisiana households living in coastal areas of southeast Louisiana, as noted above. The five waves of data were collected in June 2010, October 2010, April 2011, April 2012, and April 2013. The response rates for each wave were 20%, 24%, 25%, 20% and 19%, respectively. Items in the dataset measured community sentiment, social ties, mental and physical health, blame and distrust regarding the oil spill, financial situation, employment in industries affected by the oil spill, and demographic variables.
**Data source location**	Coastal portions of Plaquemines, Lafourche, and Terrebonne Parishes and the community of Grand Isle, Louisiana, USA. See Figure 1 below.
**Data accessibility**	Raw data and the list of corresponding survey questions for October 2010, April 2011, April 2012, and April 2013 are publicly available through the Gulf of Mexico Research Initiative Information & Data Cooperative (GRIIDC) at: https://data.gulfresearchinitiative.org/pelagos-symfony/data/Y1.x150.000%3A0002 https://data.gulfresearchinitiative.org/pelagos-symfony/data/Y1.x150.000%3A0003 https://data.gulfresearchinitiative.org/pelagos-symfony/data/Y1.x093.000%3A0001, https://data.gulfresearchinitiative.org/pelagos-symfony/data/Y1.x093.000%3A0002
**Related research article**	Cope et al. [1]. These data have also been utilized elsewhere [2], [3], [4], [5], [6].

## Value of the data

• These data help us understand the social, economic and health factors over time of coastal residents in the aftermath of the BP-DH oil spill.
• These data are helpful in furthering the work of scholars who study disaster processes, policy-makers in the Gulf Region, emergency workers, and humanitarian aid distributors.
• This repeated cross-sectional data can be used to further investigate trends in oil spill outcomes, disaster processes, health and safety factors, and economic impacts.
• These data can inform future oil spill assessment surveys in the Gulf of Mexico and the social correlates of oil spills in general.

## 1 Data description

**Fig. 1:**
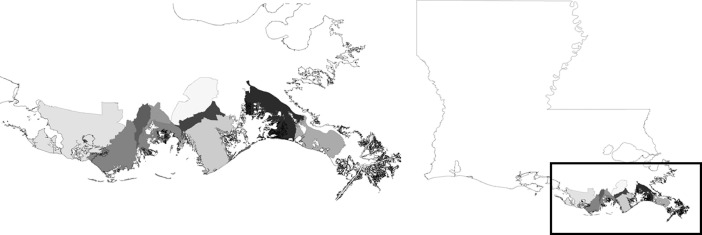
Geographic depiction of areas included in the COSS.

These data are a repeated cross-section of Louisiana households living in the coastal portions of Plaquemines Parish, Lafourche Parish, Terrebonne Parish, and the community of Grand Isle (see Fig. 1). These locations were chosen as a result of their close proximity to the BP-DH spill. The data include measures of community sentiment, social ties, mental and physical health, blame and distrust regarding the oil spill, financial situation, employment in industries affected by the oil spill, and demographic variables. Raw data and the list of corresponding survey questions included in the COSS are available for download online (see links above).

## 2 Experimental design, materials, and methods

The Louisiana State University's Public Policy Research Laboratory collected data via a telephone survey of randomly selected households from a list of approximately 6000 households in the coastal zip codes of the aforementioned areas. The zip codes were sampled from the COSS because of their geographic proximity to the BP-DH spill. The five waves of data were collected in June 2010, October 2010, April 2011, April 2012, and April 2013. The respective response rates are as follows: June 2010, 20%; October 2010, 24%; April 2011, 25%; April 2012, 20%; and April 2013, 19%. Such response rates are considered acceptable and are comparable in range to those regularly reported by leading survey organizations (e.g., Pew Research Center), despite the COSS being conducted in a disaster context and not under normal conditions [7].

The five waves of cross-sectional data allow researchers to study trends over time in the target population. The data is not longitudinal cohort data (i.e., it does not measure the same group of respondents at each time point).
